# Expert quotes and exaggeration in health news: a retrospective quantitative content analysis

**DOI:** 10.12688/wellcomeopenres.15147.2

**Published:** 2019-07-08

**Authors:** Francien G. Bossema, Peter Burger, Luke Bratton, Aimée Challenger, Rachel C. Adams, Petroc Sumner, Joop Schat, Mattijs E. Numans, Ionica Smeets

**Affiliations:** 1 Department of Science Communication and Society, Leiden University, Leiden, The Netherlands; 2 Computational Imaging, Centrum Wiskunde & Informatica, Amsterdam, The Netherlands; 3 Media Studies, Leiden University, Leiden, The Netherlands; 4 School of Psychology, Cardiff University, Cardiff, UK; 5 Department Public Health and Primary Care, Leiden University Medical Center, Leiden, The Netherlands

**Keywords:** press releases, news articles, public health, science communication, exaggeration, expert quotes, journalism

## Abstract

**Background** This research is an investigation into the role of expert quotes in health news, specifically whether news articles containing a quote from an independent expert are less often exaggerated than articles without such a quote.

**Methods** Retrospective quantitative content analysis of journal articles, press releases, and associated news articles was performed. The investigated sample are press releases on peer-reviewed health research and the associated research articles and news stories. Our sample consisted of 462 press releases and 668 news articles from the UK (2011) and 129 press releases and 185 news articles from The Netherlands (2015). We hand-coded all journal articles, press releases and news articles for correlational claims, using a well-tested codebook. The main outcome measures are types of sources that were quoted and exaggeration of correlational claims. We used counts, 2x2 tables and odds ratios to assess the relationship between presence of quotes and exaggeration of the causal claim.

**Results** Overall, 99.1% of the UK press releases and 84.5% of the Dutch press releases contain at least one quote. For the associated news articles these percentages are: 88.6% in the UK and 69.7% in the Netherlands. Authors of the study are most often quoted and only 7.5% of UK and 7.0% of Dutch news articles contained a new quote by an expert source, i.e. one not provided by the press release. The relative odds that an article without an external expert quote contains an exaggeration of causality is 2.6.

**Conclusions** The number of articles containing a quote from an independent expert is low, but articles that cite an external expert do contain less exaggeration.

## Introduction

News media coverage influences health outcomes ( Catalan-Matamoros & Peñafiel-Saiz, 2019; Matthews *et al*., 2016; Sambrook *et al*., 2010). When news media incorrectly cover new developments in medication or serious medical issues, this can have harmful effects ( “A place to tell stories of patient harm from misleading media,” n.d.; Schwitzer, 2008). Although a number of parties are involved in the production of health news, most prominently: journalists, researchers, and institutional PR staff, journalists in particular receive a major share of the criticism.

Restrictions of time and space are oft-mentioned excuses for errors in the news such as distortion or exaggeration. Journalists lack the time to do proper research and have to respect maximum word limits or air time ( Entwistle, 1995; Schwitzer, 2008). In the case of medical news, journalists may also be hampered by a lack of knowledge, as many have no specialized training in health subjects ( Tanner, 2004). Training journalists and/or cooperation with independent physicians might help to reduce the gaps in knowledge and shortcomings of medical news ( Schwitzer, 2008).

Inaccuracies in the news, however, frequently originate in the press releases journalists take their cue from ( Schat *et al*., 2018; Sumner *et al*., 2014). Press releases often present the findings in a way that increases the perceived importance and often do not mention study limitations ( Sumner *et al*., 2016; Woloshin & Schwartz, 2002). As a consequence, health news items frequently contain the same kinds of exaggeration that are already present in the press releases: unwarranted health advice, presenting non-human samples as if they were human, and correlations presented as causal relationships ( Schat *et al*., 2018; Sumner *et al*., 2014). Since the 1990s, exaggeration, or more broadly speaking, hype, caused by the blurred demarcation between science, news media, and marketing, is recognized as a systemic problem in science communication ( Weingart, 2017).

In spite of these shortcomings, press releases by universities, medical centers and scientific journals still constitute a major news source ( Autzen, 2014; Schwartz *et al*., 2012). Moreover, previous research shows that sending out a press release increases the probability that research is covered by the media ( De Semir *et al*., 1998; Schat *et al*., 2018; Schwartz *et al*., 2012).

Consulting an expert, in particular an independent one, might reasonably be expected to improve the news item’s quality. The presence of quotes by independent experts might even be a useful quality indicator for the audience, indicating that the journalist has independently sought added value. After all, journalists themselves claim they use experts as sparring partners ( Albæk, 2011) and one of health journalism’s staunchest critics lists seeking out independent sources as one of ten rating criteria ( Schwitzer, 2008). The second reason journalists quote experts - not necessarily independent ones - is a rhetorical one: journalists prefer to outsource claims to experts as these provide ‘compensatory legitimacy’ ( Albæk, 2011). The present study analyses Dutch and UK press reports on academic medical research to explore the relationship between the addition of quotes and the presence of exaggeration.

Assessing ‘exaggeration’ in health news implies a normative approach to the practices of communication professionals. Alternative perspectives, conceptualizing the news process as a sense-making effort by various parties with different interests, will be discussed in the context of the present study’s implications.

### Sourcing routines

Source use and exaggeration in health news have been the topic of numerous studies. Starting with general findings about journalist’s sourcing routines, this literature overview will then address sourcing patterns in health news, exaggeration (focusing on the causality - correlation mix-up), and the use of expert quotes. As a final point we included references to current editorial guidelines.

Regarding journalists’ source selection, one of the most robust findings is that journalists generally prefer familiar and institutional sources ( Reich, 2009); this applies as well to academic experts in the news ( Albæk, 2011). The advent of the internet- and social media, offering a wider range of easily accessible sources than before, has not changed this pattern ( Deprez & Van Leuven, 2018) and possibly even increased health journalists’ dependence on a limited number of big players, i.e. the major scientific journals ( Granado, 2011). Although the obligation to fact-check is part and parcel of journalists’ professional rhetoric, in practice this is often neglected ( Diekerhof & Bakker, 2012): journalists trust academic papers to have high quality, as these are peer reviewed ( Conrad, 1999) and as a consequence they fail to ask critical questions ( Furlan, 2017).

This limited use of sources is a specific instance of journalists’ over-reliance on prepackaged news, a practice that has been lambasted as ‘churnalism’ ( Davies, 2008; Van Hout & Van Leuven, 2016). Press releases from scientific studies are even often literally copied ( Autzen, 2014). Errors present in the press release, or in the academic paper, can thus end up in the news report ( Sumner *et al*., 2014). In this paper, we focus on one category of error: exaggeration.

### Exaggeration: causality and correlation

There are many ways in which scientific findings can be exaggerated in the media and it is a daunting task to analyse all of them. Therefore, we focus on a widespread type of exaggeration: inferring causality from correlation in health news. Many health studies are designed to detect correlations, but their results are regularly presented as causal relations - implying a mechanism that might not exist. In an analysis of the 50 most online-shared scientific health articles and their associated media articles, reviewers found that 34% of academic studies and 48% of media articles overstate how strong the evidence is that the study proved a causal relation ( Haber *et al*., 2018).

University press releases have also been shown to exaggerate correlations. A content analysis of 462 UK university press releases and their associated scientific articles and media coverage showed that 33% of the press releases contained exaggerated causal claims ( Sumner *et al*., 2014). Furthermore, there was a strong association between exaggeration in press releases and in news articles. When the press release was correct, so were 82% of the news articles, but when it was exaggerated, so were 81% of the news articles. These results have been replicated for press releases from leading peer-reviewed journals ( Sumner *et al*., 2016) and for Dutch university press releases ( Schat *et al*., 2018), leading to a similar conclusion.

### Expert quotes

Adding quotes from independent experts could help journalists avoid making mistakes. An external expert may add a more critical perspective. In fact, journalists in general routinely appeal to authority by including the voice of experts. Presenting claims in the form of quotes also warrants objectivity: this is not the journalist speaking, but the expert – although the journalist selected both the source and the quote ( Conrad, 1999; Tuchman, 1972). Press releases about health studies usually contain ready-made quotes by one of the researchers ( Woloshin *et al*., 2009). Striving for balance, journalists may ask an independent expert for a second opinion, as they can be more critical ( Schwitzer, 2008). However, when presented with stories containing hedging about the certainty of cancer research, test subjects rated both journalists and researchers as *less* trustworthy when hedging was attributed to unaffiliated experts. Self-criticism by the researchers who authored the featured study however, bolstered both their own and the journalist’s reliability ( Jensen, 2008).

Whether a story needs an extra expert quote to be balanced depends on the opinion of the journalist and their views on balance (i.e. if there is consensus in the scientific world, there is no need for a second opinion) ( Conrad, 1999). For the reader, the fact that the story contains a quote from an independent expert, indicates two things: that the journalist has spoken directly to an academic; and that the journalist has had at least some degree of time to prepare the story before writing it (or if the story is churnalism between news outlets, then at least the journalist of the original story did this). In the UK, the Science Media Centre facilitates sourcing expert quotes, which helps reduce, but does not eliminate, the extra time journalists need to integrate multiple sources into a story.

It was previously found that, in order to add a measure of human interest and to obtain better quotes than those offered by the press release, journalists may contact one of the study’s authors ( Entwistle, 1995). This was found less common in coverage of peer reviewed publications, since journalists tend to trust the reliability of this kind of source ( Entwistle, 1995; van Trigt *et al*., 1994). Journalists may also fear that a second expert, motivated by rivalry, could be less, rather than more objective. Journalists prefer to quote well-known researchers or experts who proved helpful and reliable to them in the past ( Albæk, 2011; Entwistle, 1995).

Based on interviews with 15 science reporters, Conrad (1999) found that an important reason for including a certain source is accessibility. Other important reasons were stature and quotability, i.e. the ability of the speaker to provide clear and short quotes. In science news about genetics and behavior, Conrad distinguished five uses for expert quotes: providing context, legitimation, explication, balance and outlining a study’s implications.

References to ‘independent experts’, however, may be misleading. Wang *et al.* (2017) question both the independence and the expertise of ‘independent experts’. In their sample of news stories, only 1 in 6 contained a quote by an independent expert. 25% of commenters did not possess relevant academic or clinical expertise. More than half of the comments involved academic conflicts of interest, and one third involved financial conflicts of interest. 

The literature describes journalistic routines regarding expert sources, highlighting professional practices and the rhetorical function of (expert) quotes. In Tuchman’s classic characterization, (expert) quotes are part of the ‘strategic ritual’ of objectivity ( Tuchman, 1972). Whether expert quotes actually enhance accuracy remains unexamined.

### Newspaper guidelines

In order to flesh out the academic literature on expert sources we include some written and unwritten rules from the newsroom work floor. Most newspapers in our sample have their own guidelines for dealing with press releases and the quotes they contain, although these rules are not always written down. Of the Dutch and UK newspapers we contacted, only one had an explicitly written policy regarding including independent experts in each science story ( De Volkskrantcode, 2018). However, all indicated the importance of speaking to and quoting both the academic authors and one or two independent experts.

A noteworthy example of guidelines outside the newspaper editorial offices are the Science Media Center’s ‘ *The 10 best practice guidelines for reporting science and health stories*’. These guidelines were drawn up to assist journalists in giving a balanced and accurate story but are not in any way binding. One of the guidelines states: ‘If space, quote both the researchers themselves and external sources with appropriate expertise. Be wary of scientists and press releases over-claiming for studies.’ ( Science Media Centre, 2012) A recent development is the *‘AMS press release labelling system for new medical research’* that gives press release officers a guidance on labelling their press releases with criteria such as whether the research was peer-reviewed, the type of study and the form of life that was studied. The system is ‘meant’ to help journalists see at a glance the nature and significance of new research’. It explicitly warns press release writers not to use causal language if the study type does not allow for it and advises to consult the scientists involved ( Science Media Centre, 2018).

The University of Leicester’s ‘ *Press release checklist*’ points out the responsibility of the scientists. This list includes questions on whether the press release is an accurate reflection of the scientist’s work and whether the scientist has contacted any people with an interest in the work (i.e. colleagues or funders) ( “Press Release Checklist — University of Leicester,” n.d.). Currently, Danske Universiteter, the association of Danish Universities, is working on guidelines for press releases, based on the UK and Dutch studies on exaggeration in science news ( Hoffmann, 2018). They wish press releases to describe the study design, including what type of study was done (for example, on humans or animals) and whether the results show a correlation or a causality.

### Hypotheses

Based on the literature and the guidelines outlined above, the hypotheses underlying the current research are:

H1: In both press releases and news items, the most quoted person is one of the authors.
H2: In news articles, there are more quotes by external sources than in press releases.
H3: News articles that contain a ‘new’ quote (i.e. a quote not literally taken from the press release, indicating that the journalist has had time to gather new material when preparing the story), will less often contain exaggeration than news articles that do not contain a new quote.
H4: When this ‘new quote’ is an independent (external) expert, the exaggeration will be even less frequent.

If the last hypothesis is true, we would be able to give the newspaper reader a rough guideline for the quality of an article containing information on health news, by checking who is quoted or mentioned as source. The goal of this research is therefore to find out whether we can establish an easy first check of accuracy for readers, so that they may be able to spot the more reliable articles. To our knowledge the link between quotations and accuracy of news articles has not been previously studied.

The present paper combines the results of similar research with independent samples by Cardiff University and Leiden University. This unique cross-border collaboration gives us the opportunity to test the relation between quotation and accuracy for the United Kingdom and The Netherlands combined, enlarging the tested material and strengthening the conclusions.

## Methods

From publicly accessible university repositories, the UK study identified all press releases published in 2011. To be included the press releases needed to be based on peer-reviewed studies with possible relevance for human health (biomedical and psychological sciences) by the Russell Group universities (the 20 leading UK research universities). Press releases that were not on a health related subject or not based on a peer-reviewed journal article were excluded. For each relevant press release (n=462) the associated peer reviewed journal article and print or online news stories (n=668) from the national press were sourced using the Nexis database, BBC, Reuters, and Google. News selection was based on a direct relationship to the included press releases, no other selection criteria were taken into account.

For the Dutch study, all press releases of universities and university medical centers were sourced using university websites. Inclusion criteria were that the press release must be based on one peer-reviewed scientific article, on the subject of biomedical and health-related research and published in 2015. Press releases were excluded if based on several scientific articles or if they did not deal with correlation between two variables (for example only a statement about a newly developed technique). The associated peer reviewed articles were found using database PubMed and Google Scholar. News articles were again selected based on their relationship with the selected press releases, using press database LexisNexis, Google, the websites of newspapers and news websites. In total 129 press releases and 185 news articles were included.

In both studies the number of news stories per press release ranged from 0–10. For more details on the methods we refer to Sumner *et al*. (2014) and Schat *et al*. (2018).

Sumner *et al*. used a codebook with questions on various variables of interest, including exaggeration of the causal statement and the presence of a quote in both press release and news article(s). The Dutch Schat *et al*. study used a thoroughly translated version that was very similar to this codebook. All journal articles, press releases and news articles were read and coded by one or more coders.

The main difference in the execution of the two studies was the way in which intercoder reliability was established. For the UK study the material to code was divided among 5 coders, while the Dutch team employed 2 coders. To secure intercoder reliability the UK coders double-coded 27% of press releases and journal articles and 21% of news articles. The percentage of agreement on the exaggeration variable was 89.5 and 75.4 for press releases and news articles respectively. For the presence of a quote the agreement was 99.2 and 95.8 percent. For the source of the quote in the news articles the agreement was 83.6% on average over a maximum of three quotes.

For the Dutch study both coders coded the same 10 sets (journal article, press release and news article(s)) of material, that were similar to, but not part of the research sample. Of these, the percentage agreement was tested and evaluated. After revision of the codebook a second test round of 10 sets was performed by both coders. The percentage of similarity was 90% for the exaggeration variable and 100% for the variables regarding quotes for both press release and news articles. After establishing the intercoder reliability the research sample was independently coded by the two coders.

For each press release, in the UK study as well as in the Dutch study, we collected the following information: does the press release contain a quote, and the number of associated news articles. For the news stories we collected whether the article contained a quote, whether the citations were taken literally from the press release and whether the news article was exaggerated. In both studies ‘spoken quotes’ were taken into account, i.e. literal quotes between quotation marks or when the journalist clearly writes down what someone has said (‘Mister X tells us that…’). Additionally, the type of person (author, external specialist etc.) who was cited was coded. The UK study moreover included written quotes (i.e. text literally taken from the journal article or Wikipedia). Note that quoting the journal article does not occur in Dutch newspapers, as the need to translate from the (almost always) English journals automatically induces rephrasing.

Based on the findings on the interpretation of causal statements by readers ( Adams *et al*., 2017), a five-point scale was used to measure exaggerations.

1. No relationship mentioned

2. Statement of no relationship (‘wine does not cause cancer’)

3. Correlation, ambiguous or conditional statement of relationship (‘wine associated with cancer’/ ‘wine might cause cancer’)

4. Statement of ‘can’ (‘wine can cause cancer’)

5. Statement of causation (‘wine causes cancer’).

Taking the peer reviewed paper as a baseline we sought cases where news stories made causal claims beyond (or different to) that stated in the associated peer reviewed paper.

We identified exaggeration when the code number for the press release or news article was higher than the code for the scientific article (and both contained a statement of relationship). 

### Analysis

The percentage of press releases and news articles containing a quote was calculated. We investigated what type of people are quoted by calculating the percentage of articles that contains a quote from a person in one of the categories.

In order to assess whether a news article without a new quote (a quote that is not taken literally from the press release) is more likely to contain exaggeration of the causal claim, we performed a statistical analysis using 2×2 tables to calculate the corresponding odds ratio within a 95% confidence interval using OpenEpi software ( OpenEpi, n.d.). The same was done for the correlation between quotes from external experts and exaggeration of the causal claim. An odds ratio of 1 or higher indicates that exposure to the variable of interest (the lack of a new or external expert quote) is associated with a higher risk of the investigated outcome (exaggeration) ( Szumilas, 2010).

## Results

Overall, 99.1% of the UK press releases and 84.5% of the Dutch press releases contained a quote. For the news articles 88.6% of those in the UK and 69.7% in the Netherlands contained at least one quote.

Citing sources may add nuance and clarification to a news article. An example from our sample is a study into the relationship between Parkinson’s disease and creative professions. Press release and news reports alike claimed that creative jobs protect against Parkinson’s disease. One of the researchers, upon being asked by a reporter of De Volkskrant, said that the news articles *“did not give a fully correct representation of the journal article’s results”* and that the suggested causal relation might actually be reversed. She explains that the results in some other media were over-simplified and thereby gave a false conclusion ( Barbier, 2015). This example shows that calling an expert for a quote can improve the accuracy of a news article and ensure that the results are represented in the right light. 

### Who gets quoted?

In the UK sample, 39.4% of the total number of news articles contained a new quote from one of the study’s authors. Furthermore 4.3% of the news articles directly quoted the journal article and 26.8% cited a new source that cannot be verified, e.g. Wikipedia. Only 7.5% of the news articles featured a quote from an independent expert source. Finally, 0.1% of the articles featured a quote that was irrelevant to the research article. 63.9% of the news articles copied a quote from the press release.

Of the Dutch news articles 34.6% copied a quote from the press release. For the subsequent Dutch study, we looked more in depth at the nature of the sources quoted, see Table 1 for a summary. Note that press releases and news stories may contain more than one quote, with quotes falling in different categories. So one news story can be reflected in two percentages and thus these cannot be added to get a total over different categories.

**Table 1.** Percentage of Dutch press releases and news articles that quote a certain type of source. Since some press releases and news articles contain several quotes of different types, news stories are counted in more than one of the categories and the percentages add up to more than 100%.

	Press releases	News articles
Author	82.9	63.8
Researcher of the same institute	3.1	1.6
Non-involved researcher	0	3.8
External specialist	0	4.9
Interest group	1.6	3.8
Other	0	2.2
No quote	15.5	30.3

As a non-involved researcher we took anyone who is not working at one of the research institutes of the authors of the paper. An external specialist is a non-researcher, who does have expertise in the field of study, for example a medical doctor. These two categories together form the category *external expert* that will later be used for the analysis of the relationship between external expert quotes and exaggeration.

These results confirm hypothesis 1, as the authors of the scientific paper are the most quoted in both press releases and news articles. Moreover, quotes from independent experts (UK data) non-involved researchers, external specialists and interest groups (Dutch data) are relatively rare. As can be seen in Table 1, in every case these external quotes occur more often in news articles than in press releases, confirming hypothesis 2. It is worth noting that the reason the source of the quote in UK press releases was not even coded was because they are virtually always from the study authors. 

As the previous results show the two datasets to be similar, we have combined them for the rest of the analysis. The data per country is available as Underlying data ( Bossema, 2019).

### New quotes and exaggeration

‘New’ quotes can help journalists to clarify and correctly represent a study’s results. We define a new quote as any quote that is not taken literally from the press release. This can be from the same source as a quote in the press release, but a new text, or a person not mentioned in the press release at all. It therefore indicates that the journalist spoke to at least one person before publication of their story.

In our sample we saw new quotes that clarified the difference between correlation and causality, for instance when a researcher of a study into the differences in cocaine-dependence amongst people, adds nuance to a news article in The Guardian:

*Ersche said that, though she found links between brain structure and cocaine use, her research was not conclusive on which came first. "At the moment, correlation shows me a direct relationship - but I don't know which direction the relationship is. Has this been caused by cocaine, or are people who have this abnormality more vulnerable?"* ( Jha, 2011)

However, these types of quotes were rare, and more commonly quotes summarised the findings, emphasised their importance, and/or called for more research. Concerning the role of new quotes, our results ( Table 2) do not prove any relation between new quotes and exaggeration of news articles (hypothesis 3).

**Table 2.** Total number of news articles with and without new quotes and with exaggerated causal claims or not - in total 194 news articles in the sample were not applicable (i.e., contained no statement of cause or correlation that could be tested for exaggeration). The relative odds that an article without a new quote contains an exaggeration of causality is 0.94 (95%: 0.7-1.3).

	News article exaggerated	News article not exaggerated	Total
News article without new quote	88 (26.4%)	245 (73.6%)	333
News article with new quote	90 (27.7%)	235 (72.3%)	325
Total	178	480	658

### External expert quotes and exaggeration

Quotes can be used to include an independent expert’s view on the subject and if necessary add a critical note. An example is a news article on a study that discussed the relationship between chocolate consumption and reduced risk of cardiometabolic disorders. In the BBC news article, the first quote is by one of the researchers, advising chocolate abstainers not to change their behavior. A more critical note is added by the quote of Victoria Taylor, senior heart health dietician at the British Heart Foundation:

*"Evidence does suggest chocolate might have some heart health benefits but we need to find out why that might be. We can't start advising people to eat lots of chocolate based on this research. It didn't explore what it is about chocolate that could help and if one particular type of chocolate is better than another. If you want to reduce your heart disease risk, there are much better places to start than at the bottom of a box of chocolates."* ( “Chocolate may protect the brain and heart”, 2011)

See Table 3 for the results on the relationship between exaggeration and external expert quotes (hypothesis 4). The relative odds that an article without an external expert quote contains an exaggeration of causality is 2.6 (95%: 1.1-6.3). Note that external expert quotes are rare, but articles that cite an external expert do contain less exaggeration.

**Table 3.** Total number of news articles with and without external expert quotes and exaggerated or not - in total 194 news articles of this sample were not applicable (e.g. contained no statement of cause). The relative odds that an article without an external expert quote contains an exaggeration of causality is 2.6 (95%: 1.1-6.3).

	News article exaggerated	News article not exaggerated	Total
No external expert quote	172 (28.1%)	440 (71.9%)	612
External expert quote	6 (13.0%)	40 (87.0%)	46
Total	178	480	658

## Discussion

### Normative approach

Our position in this paper is unapologetically, but not - we hope - naively normative. Within health communication and journalism studies, two complementary perspectives on health news can be distinguished ( Hallin & Briggs, 2015): the ‘linear’ approach to health news versus the perspective on health news as a sense-making practice. The ‘linear-reflectionist’ model conceptualizes the news-making process as a series of steps, with the original study as its starting point. Within this paradigm, accurately representing the original research findings is the main criterion for evaluating the products (i.e., press releases and news items). In its purest form, this model is a variation on the much-maligned but never completely abandoned deficit model of health communication.

Alternatively, health news can be understood ‘as part of a complex, multi-sited process in which researchers, clinicians, public health professionals, journalists, advocacy groups and audiences co-construct medical subjects and objects.’ ( Hallin & Briggs, 2015, p. 92) Within this paradigm, journalists can be seen as mediators, connecting actors with diverging interests (pp. 93–95), and the medical community itself as part of a network of knowledge-producing institutions in its own right. Within this alternative perspective, ‘clinical medicine, biomedical science and public health are more than bodies of knowledge about nature and how to control it. They are social institutions and cultural systems, embedded in complex sets of relationships with the state, the market, media and the structure of social stratification, deeply affected by their relationships with those structures and in turn shaping them.’ (p. 97).

Our position is closer to the linear view than a fully constructionist one, because we believe accuracy is a key criterion for science communication and we can use evidence to decide which texts exaggerate the findings of the original study and which do not. At the same time, we understand that there is no unique way to define what is right and what is exaggerated, and that the aims, methods, and results of medical research are not the only relevant measures of all things medical.

Our aims are consistent with the stated aims of press officers and journalists, who would generally agree that they are trying to operate within the bounds of what the science means, even as they try to make their stories relevant, personal, and appealing.

In the present study, we are merely operationalising some limited aspects of ‘accuracy’ for the purposes of research, acknowledging that there is no single objective way to identify ‘hype’, as there is no one ‘truth’ about what a science communication ‘ought’ to be. We use deviations from the peer-reviewed paper as a proxy measure of exaggeration, acknowledging that the peer review process is an imperfect instrument to collectively gate-keep what is appropriate to say about each piece of published evidence.

### The rarity of independent experts

The frequency of quotes by independent experts in our sample was relatively low. A mere 7.5% of UK and 7.0% of Dutch news articles contained a new quote by an expert source. This is even lower than the 1 in 6 found by Wang *et al.* (2017). Moreover, these external expert quotes could not, as we had hoped, be used as easy to spot indicators for accuracy. Like numbers in the news, they serve as rhetorical credibility markers ( Koetsenruijter, 2017). Comparing this outcome with the guidelines of newspapers, stating that an independent expert must in most cases be consulted, we conclude that there is a mismatch in what editors think is a common practice and the following of this guideline in practice.

### Quotes and exaggeration

Contrary to what we expected, we did not find evidence that news articles with a new quote that was not in the press release contain less exaggeration. When the news article contains an external expert quote that was not in the press release the odds that the results were exaggerated are less, but the number of articles with an external expert quote is relatively small. More news articles containing an external expert source would be necessary to be able to draw a firm conclusion about the role expert quotes play in the quality of a news article.

In this study we have taken the strongest causal statement in each article to be used for comparison towards exaggeration of the original statement. However, remarks that put the statement into perspective and the tone of the quotes (i.e. negative or positive) have not been taken into account. A critical quote could give nuance to the full article, thus rendering the strongest causal statement less impressive.

### Similarities and differences between the UK and The Netherlands

We use the results from comparable studies in Great Britain and the Netherlands. The results were generally consistent across countries despite some differences in the research protocol and wider differences in journalistic practice. The Cardiff sample is based on press releases by the 20 leading UK research universities. The Dutch sample covers press releases from all universities in the Netherlands. Although results are relatively consistent in both sources, there might be differences in press releases, studies and media treatment for these two groups of universities. Moreover, there might be slight differences in the coding and interpretation of the Dutch and English material, because of linguistic and cultural differences.

There are also differences in journalistic practice between the two countries. First of all, as most scientific articles are published in English, it is possible that quotes in the UK sample are literally taken from the original article. In the Netherlands these quotes would have to be translated and rephrased by the journalist. They could therefore be unrecognizable as a quote. Furthermore, in the Netherlands it is customary to let the interviewee read the news article before publication to correct errors of fact. This is considered bad practice in the UK. This means that Dutch experts potentially have more influence on how science news is reported, since they can also discuss the rest of the article with the journalist.

### Other limitations

It is not always clear from news stories whether an independent expert was contacted. Some news might even have been kept out of the media because an independent expert criticized the study, which falls out of the scope of the current research. In some cases journalists may have obtained information from external experts without including an explicit quote in the final news story. The available space in an news article may have an influence on the latter. The question arises whether articles with higher word length more often contain quotes. Although we have seen that longer articles (600 words or over) almost always contain quotes, the number of long articles is relatively low (less than 10% of the articles). Because the sample size of long articles is small, we have not investigated this relationship separately.

Furthermore, we only considered news articles based on or following a press release. Of course, there are many other ways in which a study can be brought to the attention of the press, that we did not take into account. Information sources may be different and quoting experts might have a quite different influence if the news does not originate from a press release. The press release editors play a role in the possible media attention as well. Many medical papers are not accompanied by a press release from the university. Therefore there is a discrepancy between the number of published medical papers and the number of press releases that could be linked to them. Regarding quotes, press officers may only take the effort to ask for a quote from one of the authors, when the story promises to do well in the media. In this way the articles that are attractive to the media, because of the content, could be made even more attractive by a quote. Or the quote might not be of any consequence, if the story is very newsworthy in itself. Moreover, there might be press release editors that always include a quote and ones that never include a quote, biasing the results towards certain universities.

Furthermore, this study used the statements in the scientific publications as a baseline, but many peer reviewed publications already contain exaggerations ( Haber *et al*., 2018; Lazarus *et al*., 2015; Mathieu *et al*., 2012; Shinohara *et al*., 2017). This means that the actual level of exaggeration in the media may be higher than reported and this offers another challenge for journalists, who cannot take the scientific articles at face value.

### Implications

Based on our sample, exaggeration or misrepresentation of results in health news cannot be shown to be directly related to the presence of quotes of external experts and authors. We did however perceive that exaggeration and misrepresentation are present in some press releases and news articles in our sample. The news coverage of health-related topics leaves much to be desired, but the lack of correct or complete information is not typical for this field. There are some good guidelines available, both for media and universities. These guidelines are composed to avoid some of the most common mistakes, but the questions could be more specific - for instance with checks as to whether the type of the study and the strength of the results are reported correctly. These guidelines should be followed more closely by scientists, press officers of universities and journalists alike.

We offer this advice, realizing that the actors involved in the production of health news do not necessarily strive for accurate representation of research findings. Still, we prefer to believe that accuracy - operationalized in this study as the absence of exaggeration - is valuable as an evaluation criterion among others. (For a set of potential criteria of risk reporting, see Vasterman *et al*., 2008). Journalists generally list factual and accurate reporting as their first concern, even though they may deviate from that norm in practice. Appealing to that widely supported norm, we have pointed out best practices, acknowledging that newsroom cultures and working conditions are not always conducive to the implementation of these practices.

### Future research

For future research it would be interesting to look into the cause and effect of exaggeration in news articles, as well in health-related news as in other fields. First of all it is interesting to investigate the hypothesis that a new quote reduces exaggeration. As we have not investigated the nature of the new quotes that were not present in the press release, the question arises whether the content of the quote is more important than the mere presence. The presence of a new quote suggests a more conscientious journalist but we do not see any results of reduced exaggeration from new quotes, only when this new quote comes from external experts. Is the effect from expert quotes therefore dependent on what they said? Does the quote need to mention a lack of causal relationship in order to see reduced exaggeration?

More generally it is interesting to look into what causes the news articles to contain exaggeration or misrepresentation of the results. Newsroom ethnography and interviews with press officers, scientists and journalists may yield insights into this question. For example, is it not to be expected that in the enthusiasm of publication scientists also exaggerate the importance of their results when they are interviewed by the press? And how can journalists, often covering many different topics without full training in every topic, make sure they exaggerate the least possible? How does exaggeration in health-related news influence the behaviour of patients when they go to the doctor? There are many relevant questions related to this research that we hope to be able to explore in the future.

## Conclusion

We compared the use of quotes in university press releases and associated news articles about health research in the UK and The Netherlands. We confirmed our hypothesis that the most cited source in both press releases and news articles is an author of the underlying study. We also see that external experts are cited more often in news articles than in press releases, but that a minority of news articles quotes an independent source. Contrary to what we hypothesized, news articles containing a new quote do not contain less exaggeration. News articles containing quotes from external (independent) experts do however contain less exaggeration.

## Data availability

### Source data

UK database

Figshare: InSciOut. https://doi.org/10.6084/m9.figshare.903704.v5 ( Chambers, 2014)

Dutch database

Data Archiving and Networked Services (DANS): Medisch nieuws in de media https://doi.org/10.17026/dans-z9w-h6pn ( Schat *et al*., 2017)

### Underlying data

Data Archiving and Networked Services (DANS) Expert quotes and exaggeration in health news https://doi.org/10.17026/dans-zgw-z9d3 ( Bossema, 2019)

This project contains the following underlying data:

- The Netherlands (folder containing data and analysis files for the Dutch database)
- UK (folder contain data and analysis files for the UK database)

### Extended data

Data Archiving and Networked Services (DANS) Expert quotes and exaggeration in health news https://doi.org/10.17026/dans-zgw-z9d3 ( Bossema, 2019)

This project contains the following extended data:

- Data analysis per country.pdf (continued data analysis by country)
- Research guidelines.pdf (further description of source and underlying data)
