# Supplementary material for: Expert quotes and exaggeration in health news: a retrospective quantitative content analysis
Source: Wellcome Open Res. 2019 Jul 8;4:56. Originally published 2019 Mar 27. [Version 2] doi: 10.12688/wellcomeopenres.15147.2 (PMC6619368; doi:10.12688/wellcomeopenres.15147.2)
Supplement: Peer review history

# Peer review: Reviewer response for version 2

**DOI:** 10.21956/wellcomeopenres.16767.r35936

**Published:** 2019-07-09

**Authors:** Charlotte Autzen

**Affiliations:** 1 Head of President's Office and Communications, Copenhagen Business School, Copenhagen, Denmark

By stating which communication and science communication paradigms the authors draw on in this study, it makes the implications of the study more clear to the reader. Further, by discussing their theoretical standpoint and implications of their standpoint, the results can more easily be discussed and used by other researchers.

 So, I believe the authors have addressed the comments in my first review in a very sensible manner and have no further comments to this relevant and important study.

I confirm that I have read this submission and believe that I have an appropriate level of expertise to confirm that it is of an acceptable scientific standard.

# Peer review: Reviewer response for version 2

**DOI:** 10.21956/wellcomeopenres.16767.r35937

**Published:** 2019-07-08

**Authors:** Joost Zaat

**Affiliations:** 1 Huisarts-publicist, Huisartsenmaatschappij Landauer, Purmerend, The Netherlands

All my remarks are adequately addressed.

 It is - in the revised form - a well balanced article and I have no further comments.

I confirm that I have read this submission and believe that I have an appropriate level of expertise to confirm that it is of an acceptable scientific standard.

# Peer review: Reviewer response for version 1

**DOI:** 10.21956/wellcomeopenres.16526.r35153

**Published:** 2019-04-29

**Authors:** Charlotte Autzen

**Affiliations:** 1 Head of President's Office and Communications, Copenhagen Business School, Copenhagen, Denmark

**Review**

 This study investigates the practice of using expert quotes in health news articles in the UK and the Netherlands. It analyses two independent national data sets, each consisting of organizational press releases based on peer-reviewed research articles and associated news media articles identified by the study authors. Moreover, the study focuses in particular on news stories and press releases communicating casual claims related to health research findings. Even though it is not stated explicitly in the study, exaggeration of casual claims is framed as a problem to be solved, and the study aims to test whether the presence of external expert quotes (experts not part of the study communicated) might serve as a possible indicator of trustworthiness of the content in a news article.

 By investigating academic press releases based on peer reviewed research articles from academia (e.g. universities), the study addresses an increasing yet still sparsely researched science communication practice ^1^. This makes the study interesting and relevant. The importance of understanding the role of press releases in public communication of science cannot be underestimated at a time where classic news media practices as well as organizational communication practices of research organizations are transforming rapidly, partly due to the online presence of both (see e.g. Holliman et al., 2009 ^2^).

**Worldviews and theoretical frame**

 Designed as a relatively large scale empirical study, the study is clear on methods used, but less clear regarding its theoretical framework. This makes findings less robust than they could have been. A more clearly stated theoretical foundation of the study (e.g. the authors’ understanding of central terms such as *communication, journalism *and *media use*) would make the contributions from this study clearer for the reader.

 When discussing phenomena such as *exaggeration* in health news, we need to know whether the authors understand communication as a linear process or as a sense making process. That is, are the assumptions made about communication based on a transmission view where any misconceptions at the end of the receiver is understood  to originate from errors introduced by either the sender or noise in the channel of communication. Alternatively, is communication rather understood as a sense making process where possible outcomes depend on both sender, receiver, context and worldviews? Similar considerations apply to the understanding of other contemporary phenomena in relation to science in media. For example, how do the authors understand media use in the click-bate era when the headline might be the only line people read and remember regardless of what else is explained in the rest of the article? A way to address these points and thereby strengthen the article could be to elaborate the discussion part by for instance challenging the idea that research findings can be communicated objectively and exaggeration (or hype) can be objectively identified and should be avoided ^3^.

**Improving a communication practice**

 For a study which aims to change a communication practice a clarification of the adopted theoretical standpoint is particularly important. This study suggests to add independent expert quotes to research press releases as well as news articles to avoid exaggerations of casual claims. However, if worldviews and working norms of practitioners (public information officers and journalists alike) differ considerably from the ones of the study authors, the study’s contribution to practice can be difficult to evaluate and even more difficult to implement. Take for instance the case of journalists. If journalists for pragmatic reason (e.g. limited time) often do copy-paste academic press releases without further independent research it must be critically assessed in the study when journalists answer the opposite and thereby replicate ideal journalistic norms rather than tell about their actual practice when asked. Likewise, if universities communicate research findings in a press release format for more reasons than making the public understand science via news media ^4^, an understanding and awareness of such reasons must be considered before designing a study aiming at improving press release as a science communication practice. In an early critique of the large efforts made to communicate science to the public, Trachtman ^5^ argues that the public would be often better off without all the research findings they read about in the newspapers. So, a good question to ask would be why the press release is written in the first place and thereby also why it is written as it is. Further reflections on purposes behind research press releases might therefore prove useful to explain the observed presence of internal expert quotes but only few quotes from independent sources. Could it be that research press releases imitate journalistic news values and writing style and add quotes from a limited number of voices and at the same time adhere to scientific writing norms where credit should be given to study contributors? ^6^ This makes the inclusion of independent expert quotes difficult to implement because too many people quoted in the same text makes it less attractive when journalist churn the press release into a news piece by copy-pasting it.

 To sum up the overall main point made in this review: it would be helpful if the theoretical foundation of the study was made more explicit than it is currently the case. The advantage of stating more clearly which communication and science communication paradigms the authors have based this study on, is that the underlying assumptions following such paradigms become visible. Then results could then be challenged and discussed by taking into account the premises on which they are based.

I confirm that I have read this submission and believe that I have an appropriate level of expertise to confirm that it is of an acceptable scientific standard, however I have significant reservations, as outlined above.

## References

[1] : Academic press releases caught between theory and practice. Making sense of a contested science communication practice. *PhD Thesis (unpublished), University of Southern Denmark*.2018;

[2] : Investigating science communication in the information age: Implications for public engagement and popular media. *The Open University*.2017;111-119

[3] : Investigating science communication in the information age: Implications for public engagement and popular media. *The Open University*.2017;111-119

[4] : Is There a Hype Problem in Science? If So, How Is It Addressed?.2017;1: 10.1093/oxfordhb/9780190497620.013.12 10.1093/oxfordhb/9780190497620.013.12

[5] : Science communication and public relations: beyond borders. *Science Communication, vol. 17, Handbook of Communication Science*.(Forthcoming);

[6] : The Public Understanding of Science Effort: A Critique. *Science, Technology, & Human Values*.1981;6(3) : 10.1177/016224398100600303 10-15 10.1177/016224398100600303

[7] : Bridging Science and Journalism. *Science Communication*.2014;36(4) : 10.1177/1075547014533661 479-501 10.1177/1075547014533661

# Response

**Published:** 2019-07-01

**Authors:** Francien Bossema

**Affiliations:** Centrum Wiskunde en Informatica Amsterdam, The Netherlands

We are grateful for Dr. Autzen’s review, which raises a number of important issues regarding (1) our theoretical framework and (2) the implications of our findings for health news professionals.

First, our position in this paper is unapologetically, but not - we hope - naively normative. We are aware of the ongoing debate regarding the ‘linear’ approach to health news versus the perspective on health news as a sense-making practice. Taking a more explicit stand should clarify our argument and we revised the introduction and the discussion sections accordingly. Briefly, two complementary perspectives on health news can be distinguished (Hallin and Briggs, 2015): the ‘linear’ approach to health news versus the perspective on health news as a sense-making practice. Our position is closer to the linear view than a fully constructionist one, because we believe accuracy is a key criterion for science communication and we can use evidence to decide which texts exaggerate the findings of the original study and which do not. At the same time, we understand that there is no unique way to define what is right and what is exaggerated, and that the aims, methods, and results of medical research are not the only relevant measures of all things medical.

Second, regarding the implications of this study, although we acknowledge that the actors involved in the production of health news do not necessarily strive for accurate representation of research findings, we claim that accuracy - here operationalized as non-exaggeration - is valuable as an evaluation criterion among others. (For a set of potential criteria of risk reporting, see Vasterman, Scholten & Ruigrok, 2008). Journalists generally list factual and accurate reporting as their first concern, even though they may deviate from that norm in practice. Appealing to that widely supported norm, we point out best practices (i.e., always as for a second opinion from an independent expert), realizing that newsroom cultures and working conditions are not always conducive to the implementation of these practices.

The observation that we would ‘suggest to add independent expert quotes to research press releases’ must be a mistake: we do not suggest this, as it would obviously run counter to the purpose of a press release, which is promoting the institution’s research in ready-made news formats.

**References**

Hallin, D. C., & Briggs, C. L. (2015). Transcending the medical/media opposition in research on news coverage of health and medicine. Media, Culture & Society, 37(1), 85-100.

Vasterman, P., Scholten, O., & Ruigrok, N. (2008). A model for evaluating risk reporting: The case of UMTS and fine particles. European Journal of Communication, 23(3), 319-341.

# Peer review: Reviewer response for version 1

**DOI:** 10.21956/wellcomeopenres.16526.r35155

**Published:** 2019-04-12

**Authors:** Joost Zaat

**Affiliations:** 1 Huisarts-publicist, Huisartsenmaatschappij Landauer, Purmerend, The Netherlands

The authors tried to answer the question whether quotes from researchers in press releases or quotes from external experts/researchers have an influence on articles in Dutch and UK newspapers. The question is relevant and the methodology is sound, but I have a few questions.

1. Why the difference in time frame between the Dutch and the UK ( 2015 versus  2011)?
2. It is unclear what kind of newspaper articles were selected. I can imagine that there is a difference in quoting between sections in newspapers. There is a difference between small articles in the news section of a paper (200-300 words) or more extended articles in the scientific section (>600 words). More detail of the selected articles is needed. Were the external quotes only in the more extended articles? It could be a confounder.
3. Did the authors also select press releases from technical universities like TU Twente (technical medicine)?
4. 129 press releases from the Dutch universities in a whole year seems rather few to me (2/week). Very weak Dutch medical universities publish 200-300 articles according to my former weekly searches in Pubmed. Not all publications are accompanied by press releases but I don’t believe that the 129 forms the whole cohort. How was the selection made?

I confirm that I have read this submission and believe that I have an appropriate level of expertise to confirm that it is of an acceptable scientific standard, however I have significant reservations, as outlined above.

# Response

**Published:** 2019-07-01

**Authors:** Francien Bossema

**Affiliations:** Centrum Wiskunde en Informatica Amsterdam, The Netherlands

We thank Dr. Zaat for his review report, his compliments and his questions. We will answer the questions to the best of our ability below.

1 *. Why the difference in time frame between the Dutch and the UK ( 2015 versus 2011)?*

This study is based on two previous studies. One in the UK, Sumner *et al*. (2011), and a replication study in the Netherlands from 2015 (Schat *et al*.). For the current study we have combined and reused the already coded data from both studies in order to further analyse the datasets. We have applied further analysis to the already existing collections rather than making new collections, as the collecting and processing (reading and coding) of the data is very time consuming. Combining the datasets allows for a bigger sample size and therefor stronger conclusions.

2. *It is unclear what kind of newspaper articles were selected. I can imagine that there is a difference in quoting between sections in newspapers. There is a difference between small articles in the news section of a paper (200-300 words) or more extended articles in the scientific section (>600 words). More detail of the selected articles is needed. Were the external quotes only in the more extended articles? It could be a confounder.*

News selection was purely based on relationship to press releases that fulfilled selection criteria. We selected all newspaper articles and articles from online news sources that had a direct relationship with the selected press releases (based on peer reviewed research article on health and medical topics). You are correct that the length of the article might influence the presence of an external quote, because there is more room for it in the article. We did see that most longer articles contain a quote (either from the author or an independent source). Independent expert quotes were found both in the smaller articles as in the more extended articles. The length of the articles was coded, less than 5% of the Dutch news articles and less than 10% of the UK news articles were longer than 600 words. As this is a small subset of our data we have not analysed these separately. This is a good discussion point however, thank you for the suggestion, we will include it in the discussion section in the next version of the article.

3. *Did the authors also select press releases from technical universities like TU Twente (technical medicine)?*

For the UK sample only the Russell universities were taken into account, for the Dutch sample we included all Dutch universities and university medical centres. So the technical universities were included. We point out that different universities have very different policies on sending out press releases and some of the technical universities rarely use them at all.

4. *129 press releases from the Dutch universities in a whole year seems rather few to me (2/week). Very weak Dutch medical universities publish 200-300 articles according to my former weekly searches in Pubmed. Not all publications are accompanied by press releases but I don’t believe that the 129 forms the whole cohort. How was the selection made?*

The cohort of all Dutch university press releases is indeed much larger than 129 in a year, but many of them are not about research articles, but for instance about grants, new management or policies. We went through all press releases of 2015 and excluded everything that was not about health topics. Furthermore we only included press releases that were about one recently published peer-reviewed article, which excluded many press releases on complete PhD-theses. Furthermore, we note that many studies are collaborations between different institutions and that communication officers tend to only send out press reviews for senior first or last authors in their institutes. There is a discrepancy between the number of published papers and the number of press releases, as many medical papers are not accompanied by a press release.

Finally, as regards the reproducibility of this research, all our data including the analysis and explanation of how to reproduce our results can be found here: https://doi.org/10.17026/dans-zgw-z9d3

Please let us know what information is missing to ensure reproducibility.
